# An Overview of Potential Applications of Environmentally Friendly Hybrid Polymeric Materials

**DOI:** 10.3390/polym17020252

**Published:** 2025-01-20

**Authors:** Raluca Nicoleta Darie-Niță, Stanisław Frąckowiak

**Affiliations:** 1 Physical Chemistry of Polymers Department, Petru Poni Institute of Macromolecular Chemistry, 41A Grigore Ghica Voda Alley, 700487 Iasi, Romania; 2 Faculty of Environmental Engineering, University of Science and Technology, 50-013 Wrocław, Poland; stanislaw.frackowiak@pwr.edu.pl

**Keywords:** hybrid materials, environmentally friendly, packaging, medical applications, sensors, water purification, electromagnetic shielding

## Abstract

The applications of polymeric materials are being constantly reviewed and improved. In the present world, the word hybrid, and the general idea of combining two or more inherently different approaches, designs, and materials is gaining significant attention. The area of sustainable materials with a low environmental impact is also rapidly evolving with many new discoveries, including the use of materials of a natural origin and countless combinations thereof. This review tries to summarize the current state of knowledge about hybrid polymeric materials and their applications with special attention to the materials that can be considered “environmentally friendly”. As the current application field is quite broad, the review was limited to the following topics: packaging, medical applications, sensors, water purification, and electromagnetic shielding. Furthermore, this review points out the new prospects and challenges for the use of the mentioned materials in terms of creating novel solutions with different nano and micro-materials of mostly natural and renewable origin.

## 1. Introduction

The hybrid polymer market was valued at USD 8.34 billion in 2023 and is projected to increase at a compound annual growth rate (CAGR) of 4.4% from 2024 to 2032 [1]. A key driver of this growth is the increasing focus on durable and environmentally friendly materials. As global awareness of environmental issues continues to rise, industries are prioritizing the use of materials that minimize their environmental impact throughout their life cycles. Hybrid polymers, which can reduce greenhouse gas emissions, energy consumption, and waste production compared to traditional materials, are becoming increasingly important in this context.

Regulatory efforts and consumer preferences for green products are further boosting the use of sustainable hybrid polymers in various applications from automotive components to construction materials and consumer electronics. Additionally, the expanding utilization of hybrid polymers in electronics is another considerable growth factor in the market due to their potential to confer superior insulation and protection.

The limited availability of raw materials challenges the hybrid polymers market because a stable and sufficient supply of raw materials becomes crucial due to the growing request for hybrid polymers in various industries such as automotive, construction, packaging, medical, electronics, etc. Various factors such as changes in supply chains, geopolitical issues affecting the availability of resources, and rivalry within the industries employing the same raw materials can produce potential production interruption and increased costs.

Manufacturers are implementing simplified processes and cutting-edge technologies to create hybrid polymers that minimize material waste, energy consumption, and labor costs. Breakthroughs in techniques like injection molding, extrusion, and 3D printing are enhancing the scalability of hybrid polymer production, allowing for rapid prototyping and large-scale manufacturing. By improving cost-effectiveness and scalability, companies in the hybrid polymer sector can meet the increasing demand for innovative materials across various applications, including automotive, construction, electronics, and healthcare. Embracing these advancements is essential for success in a competitive market.

The non-biodegradable segment generated USD 7.8 billion in revenue in 2023 and is designed to expand at a CAGR of approximately 4.3% through 2032. This growth is attributed to its extensive range of applications and outstanding performance characteristics. Non-biodegradable hybrid polymers are favored in situations where durability, longevity, and resistance to environmental degradation are essential, being engineered to provide outstanding thermal stability, mechanical strength, and chemical resistance, which makes them crucial for high-performance applications. Although there are concerns regarding their environmental impact, continuing research and progress are aimed at enhancing the recyclability and overall environmental profile of these polymers to ensure their long-term viability and sustainability [1].

A variety of biopolymers such as poly(lactic acid) (PLA), polyhydroxyalkanoates (PHA), polysaccharides, and plant or animal-derived proteins can be modified or combined with a multitude of natural fillers such as minerals (montmorillonite, kaolin, halloysite (HNT) nanotubes, etc.) or metallic oxides (ZnO, MgO, TiO_2_, CeO_2_, and so on) to realize environmentally friendly hybrid materials valuable in various important applications.

Bioplastic hybrid films containing both plant-derived proteins (soybean protein isolate, zein, gluten protein) and animal-derived proteins (whey protein isolate, casein, collagen, gelatin) have the potential to integrate the optimal characteristics of both specific domains, offering a promising potential to develop polymeric substitutes for petroleum-derived materials. For instance, plant-derived proteins are notable in view of their cost-efficiency and biodegradability [2]. Hybrid bioplastics comprising these proteins display enhanced physical properties, transforming them into functional candidates for various applications. Plant gluten and animal keratin, which are biodegradable proteins, have great potential to be used in ecological replacements for petroleum-derived plastics, as they can be designed to undergo controlled biodegradation [3].

Multiple articles, books, reviews, and at least one comprehensive meta-review (a review of reviews) referring to the pioneers of developing various hybrid materials, the most important types, advantages, and practical multiple applications, have been published and discussed, mainly referring to classical hybrid materials [4,5,6].

In this context, the scope of this review is to explore the advantages and challenges of different examples of environmentally friendly hybrid polymeric materials, highlighting the kinds of biopolymers or natural fillers recently used in a variety of potential applications, from packaging to medical applications, sensors, water purification, and electromagnetic shielding.

## 2. Definition and Types of Hybrid Polymeric Materials

**Figure 1:**
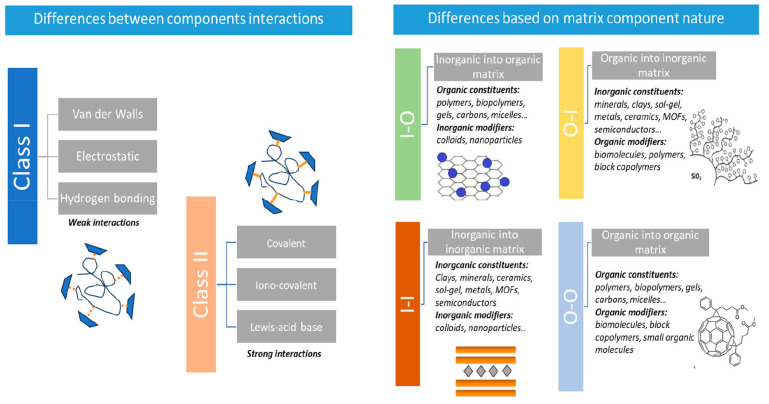
General classifications of hybrid materials, considering: the differences between the interactions of the components, class I and class II hybrid materials (**left**); the nature of the matrix and filler component, classified: I–O, O–I, I–I, and O–O types (**right**) [6]. Reprinted under Creative Common License CC-BY 4.0 of American Chemical Society.

Hybrid materials can be considered a composite type but with a very distinctive feature. They differ from traditional composites mainly in terms of scale (macroscopic for the conventional ones) and due to the more intimate interactions between the components in terms of their end properties. There are several approaches present in the current literature on how to classify such materials [7]. The most widespread classification is established by the type of relations amid the organic and inorganic components, while another takes into consideration the components as the dominant matrix and the guest (Figure 1) [6]. Most of them distinguish two classes, class I materials that possess weak interactions between the inorganic-organic components such as hydrogen bonding, electrostatic, or Van der Waals. Class II is represented by materials with strong chemical interactions between the components, mainly covalent bonds.

There are also materials described in the literature as hybrid polymer composites where the main criteria of being considered as a hybrid composite are the implementation of at least two fillers with distinctive differences in properties and/or geometry, without any specific insight into the type of bonding [8]. One of the earliest classification attempts of hybrid materials, especially polymeric ones, was proposed by Makishima [9] and referred to the following:

1. Composites: a blend of materials including matrix and micron-level dispersion.
2. Nanocomposites: a sub-micron level mixture of similar kinds of materials.
3. Hybrids: a sub-micron level mixture of different kinds of materials.
4. Nanohybrids: an atomic or molecular level mixture of various materials with chemical bonds between their diverse materials.

In some cases, the boundaries between the proposed groups are not strictly delimited and can overlap each other to some extent. I.e., nanocomposites can transpose to some extent into the hybrid materials, and so on [10]. Interestingly, the main difference or property that makes hybrid materials distinct from conventional composites is the requirement to present an atomic or at least nanometer degree of mixture. Due to the in-depth level of interactions, there is almost an infinite number of possible combinations of components (organic-inorganic), which, as a result, leads to a vast set of new materials with a very wide profile of expected properties, especially in terms of multifunctional materials with a synergistic effect of components on the materials end properties. Because of the diversity of the term “hybrid”, which is usually described as something that is “a combination” or something being a crossover with an unusual or a different set of properties in different research disciplines or industrial branches, it is necessary to limit the potential applications of these type of materials to categories as follows: packaging, medical applications, sensors, water purification, and electromagnetic shielding.

The specific applications listed here are rapidly developing in today’s research fields of polymer-based hybrid materials.

### 2.1. Hybrid Composites with Nano and Microparticles

Hybrid composites with nano- and microparticles can be obtained by various methods. And as composite preparation is not a novelty, recently there has been high interest in the formation of hybrid materials by the introduction of two or more different fillers, a preparation of the polymeric matrix using a blend of polymers, or by a combination of both approaches.

Fillers that are suitable for biomedical applications usually contain different types of inorganic materials, such as metal/metal oxide, silica, clays, graphene, graphene oxide, etc. Polymer blends are a mixture of two or more different polymers, which, by blending, offer improvement in their overall performance or required properties. Currently, many different biocomposites, including hybrid ones, are being developed to obtain materials suitable for specific biomedical applications. And as nanomaterials often exhibit superior properties when compared to conventional, micro-filler composites, they are a logical choice for an improvement in the materials science of utmost importance.

**Figure 2:**
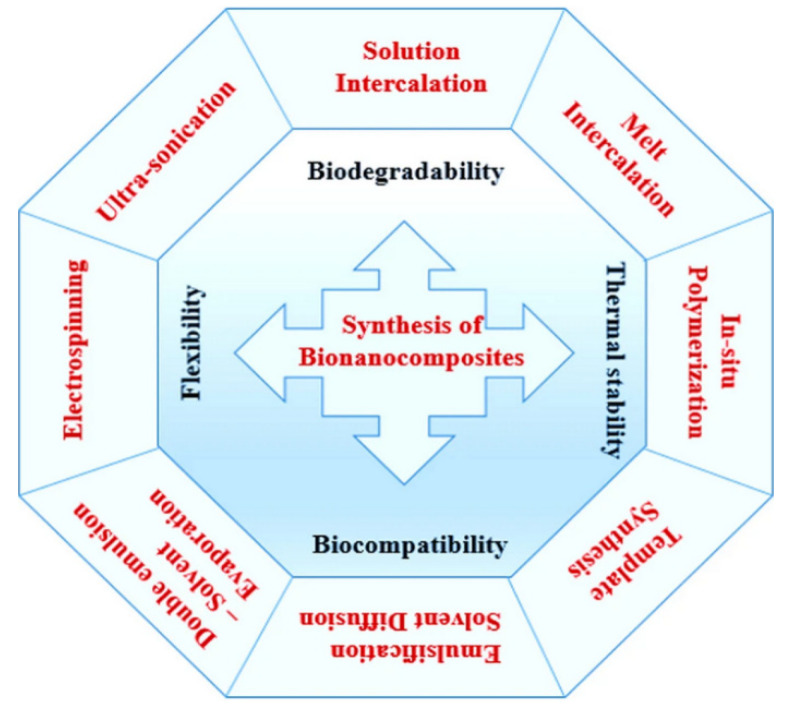
Fabrication methods of biocomposites [17].

Figure 2 presents the most available and well-known methods for the synthesis of various nanocomposites from biopolymers in regard to their required functional improvement. While there are many examples of the synthesis of the mentioned materials by, i.e., melt intercalation, in situ polymerization, and so on [11,12,13], in terms of hybrid nanomaterials, the electrospinning method strongly prevails. It is because electrospinning is an economic method for the development of continuous nanofibers by a relatively simple and easy-to-control process that allows fibers to be obtained with a specific diameter and composition, suitable for a specific application. Hybrid nanofibers can contain natural or synthetic additives such as metals, metal oxides, plant life particles, carbon-based, and others [14,15,16].

Sarıipek et al. [18] described a method of preparing a hybrid bio-based composite fabricated via a melt-spinning process. By combining silver (Ag) particles with the graphene oxide (GO), they were able to successfully obtain fibrous mats based on the poly(ε-caprolactone) (PCL) polymer with a homogenous dispersion of GO-Ag particles. And most importantly they confirmed the synergistic effect between the two fillers in the nanofiber mat samples when it comes to antibacterial activity. Another interesting example of a hybrid or better, a blend-like composite, for medical applications is presented by Kazemzadeh et al. [19] where they have successfully combined a well-known biopolymer (PCL) with polyurethane (PU). While polyurethane is not a biopolymer, it is a biocompatible material that has been used in the production of different implants for decades. By comparison of two different approaches to electrospinning, namely, co-electrospinning and the blend electrospinning method, they concluded that the prepared PCL/PU scaffolds presented improved in vivo results in comparison to co-electrospinning hybrid PCL/PU nanofibers. Furthermore, the obtained structures presented more features of improved biocompatibility than other PCL/PU scaffolds considering foreign body granulomatous reaction, tissue inflammation, and edema.

Hybrid composites containing two or more fillers were found to be of high interest in designing performant composites with properties that surpass the total of each component’s individual contribution [20,21]. Polymeric composites showing exceptional mechanical properties were realized by the incorporation of a hybrid structure of 1D/2D nanofillers, as in the case of PLA and PU-based hybrid composites, reinforced with graphene/carbon nanotubes [22,23].

Titanium dioxide (TiO_2_) is widely used as a filler in biofilms such as agar, chitosan, pullulan, wheat starch, and PLA because of its valuable properties, including non-toxicity and excellent antimicrobial and UV-blocking capabilities. The addition of TiO_2_ enhances the physical, mechanical, and thermal properties of these biofilms compared to control samples [24,25]. The hybrid material resulted from mixing lemon waste pectin with starch, and TiO_2_ nanoparticles showed biodegradability and UV-blocking capacity [26].

Another interesting group of materials that have found potential applications in many fields is a particle or fiber-reinforced polymer matrix composites. Reinforcing with clay particles has fascinated scientists due to their abundant availability, their specific layer-like structure, relatively high chemical resistance, large surface area, and water absorption ability. Clays such as montmorillonite (MMT), bentonite, sepiolite, kaolin, etc., have been widely used for the preparation of mineral-reinforced nanocomposite materials [27]. Many publications report the use of MMT containing hybrid composite materials for different applications such as flame retardants [28], dye adsorption [29], in dental [30], thermal storing [31], and other applications, i.e., biomedical [32].

The degradation of environmental quality is a pressing issue that leads to various complex problems. In response, researchers are focusing on using carbon nanotubes (CNTs) as a solution for environmental sustainability. CNTs exhibit outstanding adsorption capacity and reactivity, making them highly effective against environmental pollutants. The functionalization of CNTs has led to outstanding development in sensors and adsorbents specifically designed for environmental protection [33].

### 2.2. Aerogels and Hydrogels

**Figure 3:**
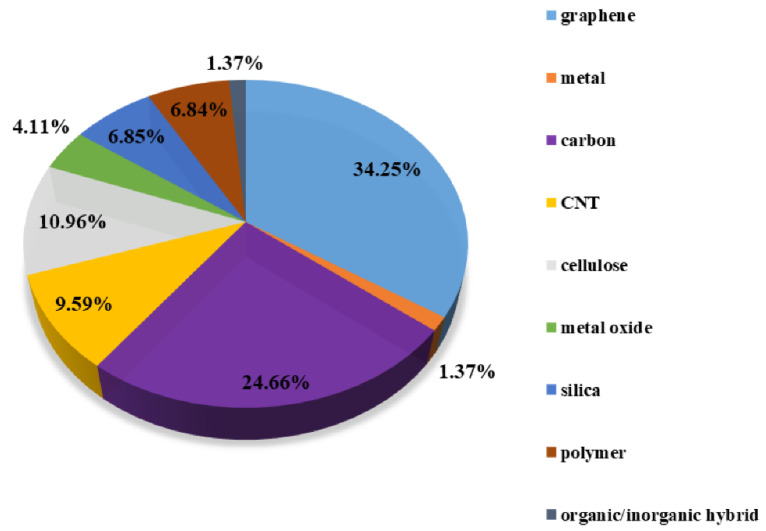
Aerogels based on chemical components. Reprinted with permission from [Jingyun Jing] (Recent Advances in the Synthesis and Application of Three-Dimensional Graphene-Based Aerogels); published by MDPI (2022) [36].

Among polymeric materials with low density, one of the most interesting types is aerogels. This group of materials can be characterized by their special properties such as extremely low density, and high porosity with large pore volume and because of the given—extremely low thermal conductivity. Such materials have been used for over a century since first developed by Kistler in 1931 [34] with the first commercial applications introduced over 20 years later. The basic principle of aerogels is a gel where the liquid is replaced by air (air content is well above 90% of the total volume). One of the lightest aerogels developed has a density of 0.18 mg/cm^3^ [35]. The potential applications are vast, they have been used in the fields of construction (thermal, sound insulation, all kinds of lightweight materials), chemical engineering (filters), civil and environmental engineering (water purification devices, absorption), electrotechnics (sensing, supercapacitors, batteries), and biomedicine (implants for tissue engineering, controlled drug release, etc.). The main types of aerogels, based on their chemical component, are presented in Figure 3.

Right now, the two main methods for producing such materials are ambient pressure drying and freeze drying, and the largest commercially available aerogels are silica-based [37,38,39]. However, biomaterials for producing such structures are also receiving a lot of attention from scientific groups, and some of the most studied precursors are alginate and chitosan [40], and other aerogels. This is because the demand for products with a possible decreased environmental impact is high, along with the promotion of possible sustainable solutions. Therefore, implementing polymers of natural origin that are carbon neutral, or close to, is becoming very important for science and commerce.

In aerogel production, materials such as polysaccharides have been widely studied and implemented, as in most cases, they provide biocompatibility and biodegradation at the same time. One other feature in favor of polysaccharides is the ability to form gels on their own (i.e., starch) in the presence of water [41]. The second group of biopolymers used for aerogel precursors are proteins, which are not as developed as polysaccharides but still provide an alternative and have been researched by numerous scientific groups all over the world [42]. To be more specific, animal- and plant-life-origin proteins such as those derived from silk, milk, soy, wheat, and others. They have proven to have a large potential for relatively easy synthesis of aerogels due to their high gel performance abilities along with good availability as they can be easily obtained from many industrial branches, especially as by-products from the food processing industry. Aerogels can also be obtained from more than one component, similar to other polymer structures. So-called hybrid aerogels have been developed for a few decades, which is connected to the fact that if their basic components can exhibit a synergistic effect, the outcome can prove to be beneficial to their properties. As with the previously mentioned biodegradability and biocompatibility, such materials have proven to have excellent potential for in vivo applications.

A similar but different material group is hydrogels. They are characterized by a three-dimensional network formed by crosslinking hydrophilic polymers through physical or chemical bonds. Among their unique properties is the ability to effectively absorb water molecules while still maintaining their mechanical and physical morphology [43]. Due to their well-developed structure, they possess superior absorption and release properties.

The development of hydrogels can be divided into three phases, called generations. First-generation hydrogels are renowned for their impressive swelling capabilities and mechanical strength, created through various chemical combinations of monomers, initiators, and crosslinkers. Chemical crosslinking enhances their properties significantly. Second-generation hydrogels, emerging in the 1970s, introduced responsive features that react to environmental stimuli. This innovation makes them essential for drug delivery and diverse biomedical applications. Third-generation hydrogels build on these advancements by focusing on interactions—both physical and chemical—between different hydrogels. This development enables the creation of complex three-dimensional materials, such as those formed from PEG-PLA interactions, paving the way for exciting future applications [44].

The structure of hydrogels, similar to conventional composite materials, consists of a polymer network (dictating the structural and other properties) filled with fluid that also adds to the mechanical properties by means of expansion and contraction.

Along with water molecules, they also absorb impurities, which makes them a common candidate for wastewater purification systems. The prime hydrogel systems for wastewater treatment are polyamides, graphene oxide, lignin, cellulose, and modified cellulose, dextran, alginate, pyrrolidone, polyaniline, polyethylene glycol, chitosan, and gelatin-based hydrogels [45]. Hybrid hydrogels present an interesting approach to combining the biocompatibility of natural polymers along with the sometimes-superior mechanical properties and versatility of their synthetic counterparts. Most of the biopolymers, among other properties, present good hydrophilic properties also granting the possibility of modifying them with different molecules, biological compounds, and so on. These combined make hybrid hydrogels a suitable candidate in the field of medical applications [46].

### 2.3. Biohybrid Materials

The arrangement of living cells with non-active materials is known as biohybrid materials, which represents a new approach to designing smart materials. Artificial materials provide protection and serve as a substrate for cells. In contrast, living cells perform various functions such as sensing, synthesis, and secretion, which can modify the physicochemical properties of composite materials [47]. By joining the advantages of both cells and synthetic materials, biohybrid materials are created, which allows living cells to carry out specific functions and promote the assembly of artificial biological systems. This innovation has greatly improved synthetic biology and materials chemistry, significantly advancing the biomedical field [48].

Biohybrid materials provide encouraging strategies to address current challenges in the biomedical field due to their exceptional biocompatibility and programmability. A variety of living cells have been explored for the development of biohybrid materials, including mammalian cells, insect cells, and microbial cells [47]. Among synthetic materials, polymers, nanoparticles, and other functional components have been utilized, ensuring their biocompatibility and mechanical properties are suitable for integration with the cells. These materials can serve various purposes, such as coatings on cell surfaces, microcarriers for cell growth, and scaffolds for organizing cells. To achieve precise control over the structure of biohybrid materials, significant efforts have been put into various fabrication methods, including molding, electrospinning, microfluidics, and 3D printing, among others. Notably, microfluidics and 3D printing play crucial roles in the production of biohybrid materials due to their ease of operation and flexibility in controlling product architecture. Using these methods, biohybrid materials can be processed into diverse configurations, such as microgels, microparticles or capsules, fibers, films, and scaffolds.

The biocompatibility and porous structure are essential for facilitating the translation of gases, nutrients, and metabolic substances [49]. To achieve these requirements, various cytocompatible materials such as collagen, sodium alginate, gelatin, silk fibroin, and agarose are commonly used in biohybrid materials [50]. Additionally, the inherent properties of non-living components, particularly their stimuli-responsive characteristics, enhance the manipulability of biohybrid materials [51].

### 2.4. Porous Structures

Hybrid composites with a specifically designed porous structure rely on the previously listed benefits of hybrid polymer-containing nanoparticles. By synergistically combining two or more materials, we are able to design and develop fine-tuned materials with a new set of chemical, optical, and, i.e., electrical properties [36]. Biopolymers have a fine place in this material group as most of them are biocompatible as mentioned before, and therefore suitable for use “in vivo”. Such materials have been extensively studied over the past years, and the emerging applications in the medical field are very promising. Porous structure materials are mainly developed for controlled drug release [52,53,54] and tissue engineering [55,56,57,58]. In tissue engineering, different approaches include extracellular matrices and hydrogels, being an alternative to conventional surgical procedures for restoring or replacing damaged organs or tissues. There are many methods available for manufacturing of such structures, namely, phase separation [59,60], gas forming [61,62], freeze drying [63], and additive manufacturing [64,65]. Developed constructs must provide accommodation for cells, allow their interaction with the surrounding environment, provide stable and sometimes complex structures to allow tissue growth with desirable stability, and release drugs in a controllable manner with reduced side effects.

Polymeric scaffolds produced from a biopolymer with a hybrid approach were developed by Ortega et al. [66]. They implemented a conventional printer for an additive manufacturing method to study the possible values of specific print parameters that could help in generating supporting structures of PCL for hybrid constructs while minimizing the print time. The primary candidates that have a direct influence on the PCL flow rate were print temperature, nozzle shape and diameter, carriage speed, and inlet pressure. They concluded via a set of experiments that it is possible to nominate several print parameters, and by doing so, they were able to greatly reduce the printing time of obtained scaffolds. Another interesting example of implementing biopolymers into tissue engineering by producing developing porous hybrid structures by combining multiple components into a single-bone scaffold, including hydroxyapatite nanoparticles (HAp), alginate, polyvinyl alcohol (PVA), and gelatine with different gelatine/PVA composition ratios, was presented by El-Bahrawy et al. [56]. HAp nanoparticles were synthesized in situ in a PVA solution, and scaffolds were fabricated using a freeze-drying method. The obtained scaffolds presented the desired properties for bone defect repair. The porosity exceeded 60%, and the swelling rate was more than 760%, along with the degradation exceeding 28 days. Scaffolds are similar in properties to the porous bone structure in terms of the stress-strain performance and absorbance of mechanical energy, which is extremely beneficial due to the fact that they can share the load with the bone tissue, thusly shielding the stress and preventing the failure of the implants. Because scaffolds developed from biopolymers undergoing degradation need to remain at their full mechanical strength for a given period of time, this issue needs to be addressed. Kankala et al. [67] have investigated the potential of porous gelatine/n-HA/PLGA scaffolds suitable for use in bone tissue engineering.

**Figure 4:**
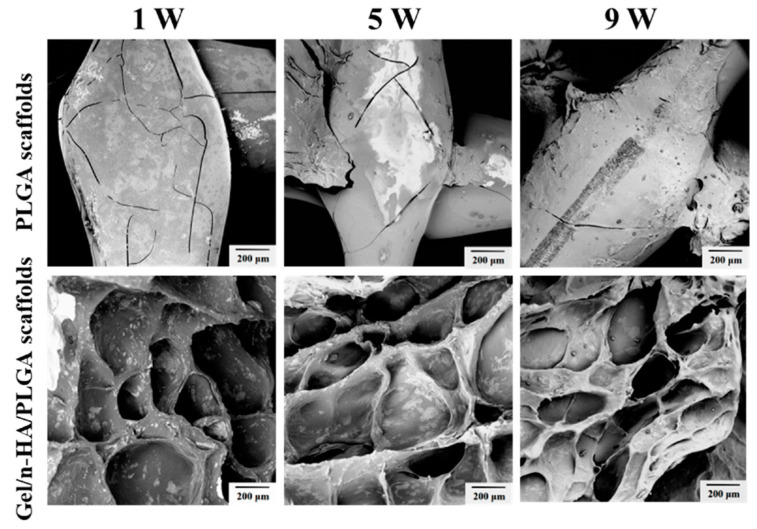
Biodegradation behavior of various scaffolds in physiological fluids. SEM images representing the biodegradation behavior of various scaffolds (the 3D PLGA scaffolds and the gelatine/n-HA/PLGA scaffolds) exposed at different time intervals (1, 5, 9 weeks). Reprinted with permission from [Kankala et al.] (3D-Printing of Microfibrous Porous Scaffolds Based on Hybrid Approaches for Bone Tissue Engineering); published by MDPI (2018) [67].

The obtained scaffolds prepared in this experiment overcame the shortcomings of single-material scaffolds. Moreover, the Gel/n-HA/PLGA scaffolds exhibited good hydrophilicity, biocompatibility, and osteogenic characteristics and were shown to produce no toxic substances during the degradation process (Figure 4).

For soft tissue such as, i.e., cartilage, similar structures are also being developed. However, it needs to be noted that for such applications, the scaffolds need to provide relatively high mechanical strength with a well-controlled pore structure for functional tissue regeneration and to allow suitable microenvironments for functional tissue regeneration. Cartilage scaffolds were prepared by hybridization of PLGA mesh and collagen using ice particulates as porogen templates by Putri et al. [57]. By using uniformly sized ice particulates as porogen templates to control the bulk pore structure of the scaffolds, homogeneous pore structures with good interconnectivity could be formed. PLGA mesh provided the enhancement of the mechanical properties of the hybrid scaffolds. In vivo implanted scaffolds presented the desired mechanical strength and a structure that can be characterized as a controlled porous one with open surface pores and also with interconnected bulk pores. The distribution of bovine articular chondrocytes and cartilaginous matrices is proven to be homogenous, which promotes high cell proliferation, cartilaginous gene expression, and high mechanical tissue cartilage-like properties.

The possibilities and combinations of different building blocks to develop a structure with a desired set of properties for medical applications are almost limitless. Apart from different tissue engineering, materials with other, more exotic properties are also being researched. Horvat et al. [68] have prepared pectin-PLA aerogels, incorporated with two nonsteroidal anti-inflammatory drugs, and with oxygen-generating agents, sodium percarbonate and calcium peroxide. As reported, it was a unique, straightforward method to produce pectin-PLA hybrid materials with a cotton-like structure applicable in wound dressings, and the prepared hybrid materials can incorporate various pharmaceuticals, independent of their water solubility.

## 3. Packaging Applications

To produce packaging systems with beneficial properties suitable for industrial applications, various materials from biodegradable polymers have often been modified by the addition of various reinforcement agents. These types of films may include mixtures of polymers, hybrid films, plasticizers, and/or nanoparticles (NPs) or nanotubes. The development of attractive, active, and intelligent packaging that leads to increasing the shelf-life of food can be achieved by the incorporation of antioxidants, antimicrobial agents, nutrients, or color-change indicators in the form of essential oils, phenolic compounds, and plant extracts into biopolymers.

Among the biopolymers, various scientific researchers have proved that polysaccharides are very suitable for food packaging, and being used for film formation or coatings, as they tend to form strong and continuous network-like structures. As neat polysaccharides do not present themselves as desirable properties for all packaging applications, alternative materials could be obtained by blending or combining with other components in hybrid materials so that the multifunctional properties necessary for packaging requirements can be attained.

Due to their properties, such as renewability, the barrier against moisture and gases, improved mechanical properties, non-toxicity, and biodegradability, potential biopolymers highly used in food packaging include carbohydrates such as starch, cellulose, agar, and other polysaccharide-based biopolymers such as chitin, alginates, pectin, guar gum, and animal-protein-based biopolymers like gelatin and collagen [69].

New active sustainable hybrid packaging materials have been developed, including proteins, polysaccharides, and lipids, in which proteins are extensively used for their outstanding gel film-forming properties [70], Some examples along with obtaining methods and their main features are displayed in Table 1.

**Table 1:** Examples of environmentally friendly hybrid materials used in food packaging applications.

Materials	Obtaining Method	Main Properties	Reference
*Polyester-based hybrid materials*			
PLA/3 wt% nano-zinc oxide (ZnO NPs) and pomegranate peel extract (PEE) (0.5, 1, 1.5, and 2 wt%)	Solvent casting	higher UV barrier, water vapor permeability and elongation at break, lower tensile strength and transparency; addition of ZnO NPs and PEE hamper the development of *S. aureus* and *E. coli*.	[71]
PLA/(1, 2, 3, 4 wt%) Magnesium Oxide (MgO) nanoparticles (NP) bio/nanocomposites films	Solvent casting	uniform distribution of MgO NPs led to improved mechanical, antimicrobial, UV screening, and gas barrier properties	[72]
plasticized PLA/0.5, 1, and 1.5 wt% Cu doped ZnO functionalized with Ag NP	Melt blending	all materials showed overall migration into three food simulants < 10 mg·dm^−2^ (accepted value according to EU Regulation No 10/2011); PLA/ZnO:Cu/Ag 0.5 showed antibacterial activity, proper mechanical and thermal properties, good barrier properties to UV light, water vapor, oxygen, and carbon dioxide	[73]
PLA/thymol (6 and 8 wt%) and 1 wt% Ag-NPs	Twin screw melt Extrusion	ternary system PLA/1 wt% Ag-NPs/8 wt% thymol showed combined antioxidant and antibacterial performance, with controlled release of thymol	[74]
PLA/acetylated cellulose nanocrystals (ACNC) (1 wt%) and ZnO nanoparticles (1, 3, 5 and 7 wt%)	Solvent casting	surface acetylation of cellulose nanocrystals improved its dispersion in the PLA; improved UV blocking, mechanical strength, oxygen, and water vapor barrier; excellent antibacterial activity against *E. coli* and *S. aureus*; migration amounts of Zn^2+^ from PLA/ACNC/ZnO film to food simulants < the specific migration limit (5 mg/kg)	[75]
PBAT/PLA/CaCO_3_ 70% polymer (where PBAT:PLA = 7:3), 30% CaCO_3_, and an additional 0.45% chain extender ADR	Twin screw melt extrusion	uniform dispersion of nano-CaCO_3_ in PBAT/PLA matrix through solid-state shear-milling (S3M) technology; film obtained by milling only PBAT and CaCO_3_ exhibited the best performance, with its longitudinal tensile strength of 22 MPa and fracture elongation of 437%	[76]
poly(3-hydroxybutyrate-co-3-hydroxyvalerate) (PHBV)/0–1 wt% Fe doped ZnO nanoparticles (NPs) deposited onto PLA film	Electrospinning/electrospraying	“beads” morphology; migration in food simulants within the legislation limits; PLA/PHBV/ZnO: Fe0.3 electrospun nanosystem showed a remarkable antimicrobial effect against *P. aeruginosa*	[77]
*Polysaccharide-based hybrid materials*			
starch/1%, 2%, and 3%, *v*/*w* cinnamon essential oil (CEO)/0%, 1%, 3%, and 5%, *w*/*w* TiO_2_ NPs	Solvent casting	TiO_2_-NPs improved functional properties of sago starch films activated by cinnamon EO; increasing TiO_2_-NP amount decreased barrier properties; Improved mechanical properties and antimicrobial activity against *E. coli*, *S. typhimurium*, and *S. aureus*; Potential active food packaging material for fresh pistachio	[78]
pullulan/chitin nanofibers (PCN) containing curcumin (CR) and anthocyanins (ATH)	Electrospinning	antioxidant and antimicrobial activities; color change with pH	[79]
pullulan/polyvinyl alcohol (PUL/PVA) nanofibers incorporated with thymol-loaded porphyrin metal-organic framework nanoparticles (THY@PCN-224 NPs)	PCN-224 synthesized via solvothermal method; encapsulation of thymol by physical adsorption method; THY@PCN/PUL/PVA nanofibers obtained by blending electrospinning	THY@PCN/PUL/PVA nanofibers showed synergistic antibacterial activities against *E. coli* (~99%) and *S. aureus* (~98%) under light irradiation. The cell viability assays and fruit preservation tests showed high film biosafety	[80]
bi-layer films incorporated with liposomes @anthocyanin/carrageenan/agar	Encapsulation/Layer-by-layer assembly (solution casting)	free anthocyanins and anthocyanin-loaded liposomes were added to carrageenan as the sensor layer of the bi-layer films; agar—used as the outer protective layer; the films with liposomes—a positive effect on the stability of the indicator films in high-humidity environments but slightly decreased the pH and ammonia sensitivity; the bi-layer film can be used as an indicator of meat freshness (shrimps); the encapsulation of anthocyanins by liposomes delayed the sensitivity of the film	[81]
*Protein-* *based hybrid materials*			
hybrid biofilms flaxseed mucilage (FSM), elastin/collagen (ELN/COL) matrix, and silk sericin (SS)	Solvent casting	SS addition increased the biofilm thickness (0.14–0.30 mm), opacity (7.02–8.51 mm^−1^), and the tensile strength (4.90–15.55 MPa) while reducing the values of moisture content (24.15–16.66%), water solubility (84.21–41.66%) and elongation at break (177.19–46.58%); enhanced thermal; in vitro antibacterial activity against *S. aureus*, *S. pyogen*, *E. coli*, and *P. aeruginosa*	[82]
flaxseed mucilage/pectin impregnated with titanium dioxide and calcium chloride	Solvent casting	addition of 5 wt% TiO_2_ enhanced UV barrier property and increased its crystallinity (from 43.6%. to 54.1%); crosslinking agent and TiO_2_ resulted in a prolonged period of biodegradation of >21 days when compared to neat film; no surface cracks and agglomerates observed; the film with 1 wt% TiO_2_ and 5 wt% CaCl_2_ could be used as fully biodegradable sandwich layer in dry food or medium water activity food requiring light protection	[69]
soy protein/cellulose nanocrystals (CNCs) and Cedrus deodara pine needle extract (PNE) (2.5, 5, 10%, *w*/*w*, based on the dry weight of SPI) films	Solvent casting	addition of CNCs decreased the moisture content of the films by disrupting the hydrogen bonds between N-H groups of soy proteins and water molecules; decrease in elongation at break, increase in tensile strength; 5–10% PNE led to a decrease in water vapor permeability; phenolic compounds from the PNE-added films conferred strong antioxidant activities	[83]
whey protein isolate (WPI)/chitosan nanofiber (CSNF) (3–6% based on the dry weight of WPI)/nano-formulated cinnamon oil (CiEO) (both emulsified and Nanostructured lipid carriers (NLC) form)	CiEO-loaded NLCs prepared by the hot homogenization sonication method; Bio-nanocomposite films—solution casting	homogenous distribution of CSNF improved mechanical properties and water vapor permeability; addition of CiEO led to a decrease in the water content, water solubility, and a slight increase in the water vapor permeability of the films; plasticizing effect of emulsified and NLC form of CiEO on the WPI-chitosan nanofiber film	[84]
hybrid films comprised of both wheat gluten and wool keratin proteins	tyrosine-mediated photo-crosslinking method; 3D printing	neutron scattering showed the presence of both hydrophobic and hydrophilic nanodomains, gliadin nanoclusters, and interconnected micropores in the matrix; increase in keratin amount led to softer hybrid films, consistent reduction in water resistivity, increased micropore size (from 1.2 to 2.2 µm), and hydrolytic degradability; 10-layer 3D-printed film showed over 92% accuracy	[85]

Different polymers with versatile properties are attractive for potential uses in food packaging [86]. Addition of various nanofillers is often used to enhance the tensile and barrier properties of biopolymer-based packaging films [87,88]. Among the various nanofillers, layered silicate nanoclays such as sodium montmorillonite (MMT) and halloysite (HNT) nanotubes are the most promising, being considered natural and sustainable materials, and their use can help in reducing the environmental impact of food packaging as they can be easily incorporated in biodegradable matrices [89]. The antimicrobial activity with a clear, effective inhibition zone against food-pathogenic bacteria such as *Escherichia coli*, *Salmonella enterica*, *Staphylococcus aureus*, and *Listeria monocytogenes* of a chitosan and polyvinyl alcohol-based composite film incorporated with ZnO nanoparticles encapsulated into HNT clay was demonstrated by Giannakas et al. (2022) as new hybrid films intended to be used in active food packaging [90].

Abdullah et al. realized biodegradable and water-resistant PVA-based hybrid nanomaterials incorporating starch, glycerol, and HNT for sustainable food packaging [91]. Due to their multifunctionality, HNTs can have other potential applications in the food industry, in addition to active food packaging, being used as a carrier for antimicrobial and antioxidant agents and food additives, such as vitamins, flavors, and colors, or can be used as a coating for food products to provide a protective layer that prevents spoilage and extends shelf-life [92].

**Figure 5:**
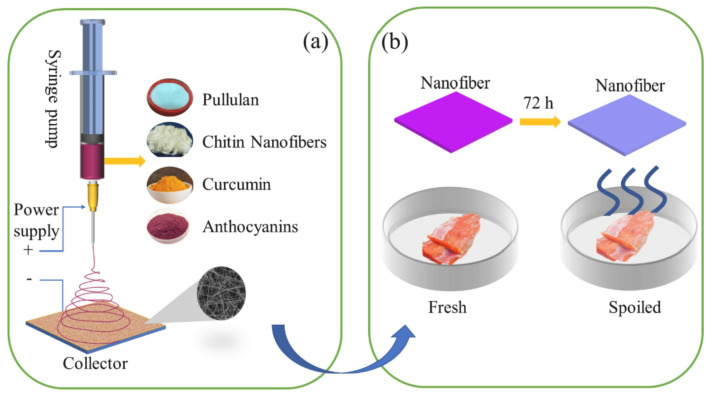
The preparation process of the PCN/CR/ATH nanofibers by electrospinning (**a**); the principle of the PCN/CR/ATH nanofiber in monitoring food freshness (**b**). Reprinted with permission from Elsevier 2024 [79].

Although pullulan, a microbial polysaccharide, is a biopolymer with biocompatibility, water-solubility, edibility, biodegradability, nontoxicity, and nanofiber-forming properties [80], pure pullulan nanofibers possess poor mechanical properties. Their properties are generally improved by the addition of nanofillers, such as chitin nanofibers used as a reinforcing agent with high mechanical strength [93]. Duan et al. developed electrospun nanofiber-based on pullulan/chitin nanofibers (PCN) containing curcumin (CR) and anthocyanins (ATH) for potential application in active-intelligent food packaging (Figure 5). The nanofiber was tested to monitor the quality change in *Plectorhynchus cinctus* at room temperature within 72 h. At the end of testing, the surface of the *Plectorhynchus cinctus* was reddish-brown, with a soft texture and a very strong odor. As the PCN/CR/ATH nanofiber changed its color from pink to powder blue, indicating a pH of 6–7 around the *Plectorhynchus cinctus*, the developed nanofiber can be used as a pH indicator for food quality monitoring because of its high pH sensitivity [79].

Agro-based residues could be valorized in producing fully bio-based hybrid polymeric materials [94]. Biodegradable films were developed by using lignocellulosic fibers from agro-based residues, with Samaniego-Aguilar et al. incorporating up to 30% micronized almond shell (AS) and Oryzite^®^ (OR) into PHBV [95]. They concluded that the thermoformability of PHBV was improved by the addition of fibers, at lower AS fiber contents or 20 wt% of OR fibers. The bio-disintegration tests showed that eco-friendly hybrid materials proved to have faster degradation during composting when including 20% and 30% AS, with great potential to be integrated into biocircularity systems.

Pomelo peel waste has been reported to be a very accessible and easy-to-collect agricultural waste that can be used as a carbon precursor. Carbon dots (CD) are considered a novel family of carbon materials, useful as nanofillers in active packaging, due to their surface functional groups and carbonaceous core and other advantages, such as excellent biocompatibility, protection against UV radiation, active properties, low toxicity, and simple synthesis methods [96]. Among the various carbon sources used to prepare CDs, the natural bioresources were of great interest due to their abundance, sustainability, and low cost. An eco-friendly material made from a biopolymeric matrix based on fish scale gelatin/alginate dialdehyde containing up to 3 wt% pomelo peel-derived carbon dots (PCDs) synthesized through a hydrothermal method has been developed and tested for its efficiency as active food packaging [97]. The incorporation of PCDs improved some important properties of composite films, namely, the mechanical performance, water-vapor resistance, UV-light blocking effects, and fluorescence properties, along with the antioxidant and antimicrobial activities. Due to these features, the multifunctional hybrid composite film containing 3 wt% PCDs extended the shelf-life of strawberries to 7 days at room temperature, preserving their physiological qualities post-harvest.

## 4. Medical Applications

Materials for medical applications are generally considered a specific group, especially when it comes to polymeric materials. Hybrid materials are versatile, due to the combination of the properties of organic and inorganic phases; the latter being nontoxic and biocompatible and, therefore, appropriate for several different applications within the medical field, namely, tissue engineering, controlled/local drug delivery, dental, and bioimaging.

Obtaining desired properties can be difficult when considering one material type (metal, ceramic, polymer) [98,99,100]. Requirements, such as mechanical strength, electrical conductivity, and toughness, combined with more biological-related properties such as general biocompatibility, types of interactions with the living tissue, cells, enzymatic reactivity, morphology, and other tailored for specific applications, can often be fulfilled only by combining one or more material groups into a single hybrid construct. Another important factor regarding the mentioned applications is the desired size on which the end products should be operating. The structure needs to be in the range of nano/micrometers in order for the end products to interact with the intended environment [100,101,102,103].

**Figure 6:**
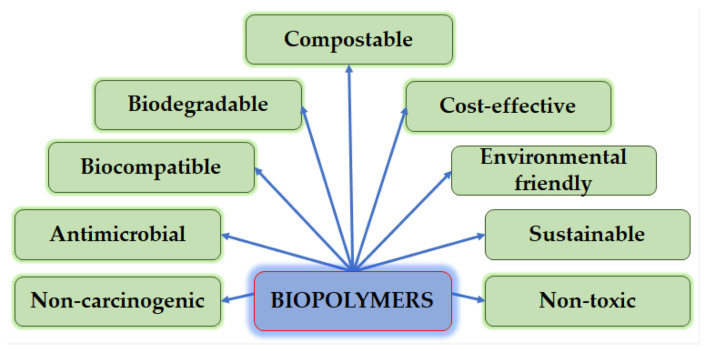
Main characteristics of biopolymers. Reprinted with permission from [Ocsana Opriș] (An Overview of Biopolymers for Drug Delivery Applications); published by MDPI (2024) [108].

Regarding the environment, the development of products for different industry sectors (e.g., packaging, construction, medicine, environmental engineering, and others), which includes biodegradable polymers, has been in the scope of many scientific groups for the last few decades. This property is a natural consequence in the history of biodegradable plastics development as most of them were first developed in the second half of the XX century for medical, in vivo implants [104,105,106]. It was not due to their biodegradability, as back then, people were not considering newly developed plastic materials as a threat due to their pollution potential, but rather it was due to their general biocompatibility and other useful features, as presented in Figure 6. I.e., lactic acid is a naturally occurring compound in the human body, not triggering the immune system, and therefore poly(lactic acid) was one of the first major polymeric materials used for internal implants [107].

Tissue engineering basically refers to the assisted regeneration of different tissues by various means and approaches. Tissues include skin, heart, bone, and others. Usual approaches consider all kinds of scaffolds made of biocompatible polymers. One of the most widely researched polymers for such applications is polycaprolactone (PCL) due to its inherent biocompatibility, biodegradability, structural stability, and mechanical properties [109]. Pantic et al. [110] developed a hybrid PCL-starch scaffold using both xerogels and aerogels. Xerogels are a porous, structural material that can be obtained via the evaporative drying of any precursor’s wet gel, with an unconstrained shrinkage. They can be characterized by a relatively less difficult fabrication, good mechanical stability, and elevated density when compared with aerogels. While starch xerogels improved interactions between the PCL and growth factors, starch aerogels improved the potential uses of PCL for bone regeneration significantly. Mesoporous-macroporous materials were obtained by combining PCL foams and chitosan aerogel beads. They concluded that hybrid materials have a higher potential for tissue engineering applications, e.g., bone regeneration applications, regarding their textural properties (pore sizes). Pore size for bone tissue scaffolds is of utmost importance as it guarantees the transport of fluids such as nutrients, metabolites, and wastes with the simultaneous accessibility of cells for bone ingrowth. Another interesting approach for developing scaffolds for bone tissue regeneration is using the electrospinning method for producing fibers and similar structures used for enhancing the desired properties [16,111,112]. Cheng et al. [40] proposed silk fibroin in the form of a solution mixed with chitin in several ratios, then underwent the process of electrospinning to manufacture the hybrid nanofibers. Secondly, the obtained nanofibers were implemented into the silk fibroin-based aerogel composites to further improve their mechanical properties. It was concluded based on the results obtained that the developed materials had improved osteogenic potential that promotes them in the field of bone defect repairing and other applications connected with regenerative medicine.

Apart from bone tissue regeneration, soft tissues like skin, for example, in wound healing applications, are in the scope of interest as well. Hybrid aerogels formed from chitosan and alginate, two naturally available polymers with beneficial physicochemical and biological properties, were proposed by Batista et al. [113]. They used a rather conventional method for the aerogel preparation, gelatinization, and subsequent supercritical drying. Although it was stated that the obtained materials were still in need of further development, they have found them to be attractive candidates for wound healing applications. Especially due to their high bioactivity.

Hydrogels are used extensively in tissue engineering applications for their unique properties that predestined them in this field, namely, high water holding capacity with physio-mechanical characteristics similar to those of natural tissue [114]. However, in recent years, there have been attempts made to introduce hybrid hydrogels as a form of overcoming the drawbacks of the original hydrogels with some value-added materials.

Lignin is known for its antibacterial and antioxidant properties and, therefore, has been widely applied in wound healing [115] combined with natural (chitosan, gelatin, agarose, carrageenan) or synthetic (polyvinyl alcohol) polymers in the form of hydrogels [116,117,118,119].

Innovative multifunctional hydrogels comprising cellulose and modified lignin (CLE) have been designed and characterized for their potential use as possible oral dressings in wound management. Ciolacu et al. concluded that the hydrogels containing CLE are biocompatible, possess muco-adhesiveness and high swelling capacity, as well as antibacterial properties against Escherichia coli and Staphylococcus aureus, being also able to release procaine hydrochloride (PrHy) in a controlled manner [120].

**Figure 7:**
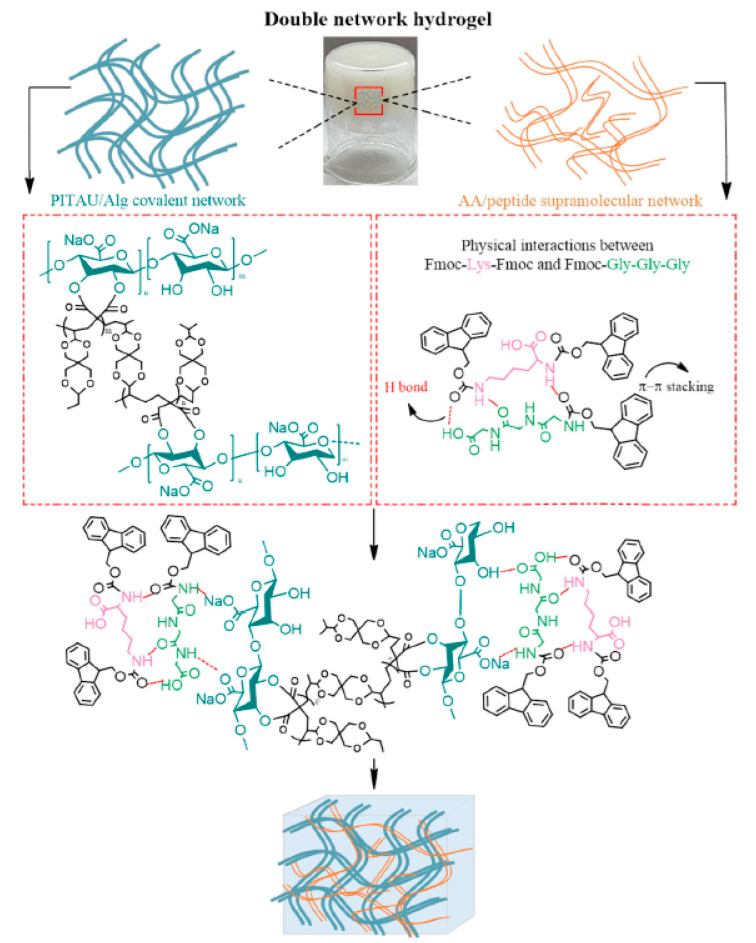
Scheme of the DN hydrogel formation comprising PITAU/Alg covalent gel as the first network and self-assembled peptides as the second network. Reprinted with permission from [Ghilan A.] (Injectable Networks Based on a Hybrid Synthetic/Natural Polymer Gel and Self-Assembling Peptides Functioning as Reinforcing Fillers.); published by MDPI (2023) [121].

Ghilan et al. [121] developed a hybrid synthetic/natural polymer gel that serves as the initial network, utilizing sodium alginate and a copolymer known as poly(itaconic anhydrideco-3,9-divinyl-2,4,8,10-tetraoxaspiro (5,5) undecane) (PITAU), which is able to form networks through specific functional groups; it is biodegradable and biocompatible, possessing also binding properties, amphiphilicity, and thermal stability, sensitivity to pH and temperature [122]. They incorporated amino acids and peptide-derived hydrogelators, like Fmoc-Lys-Fmoc-OH and Fmoc-Gly-Gly-Gly-OH, into the existing network, resulting in crosslinked double networks (DNs) through non-covalent interactions (Figure 7). The developed hybrid materials possess excellent water absorption capacity, classifying them as superabsorbent gels. The rheological properties revealed that the prepared materials exhibit injectability and self-healing capabilities. DN hydrogels showed non-toxicity and cytocompatibility, allowing the proposed compounds to be suitable alternatives for the controlled delivery of therapeutics or tissue engineering.

Bio-based porous hydrogels comprising plant extracts rich in phytocompounds have attracted great interest, mostly in healthcare areas. A one-pot ice-templating strategy has been used by Platon et al. to develop novel macroporous hybrid cryogels based on a thiourea-containing chitosan (CSTU) derivative and a *Hypericum perforatum L.* extract (HYPE), (St John’s wort). Due to the strong interactions between the functional groups of polyphenols in the HYPE and CSTU matrix, the hybrid cryogels showed high liquid absorption capacity, mechanical strength, and antioxidant and antibacterial capacities [123].

Combining different polymers, especially of natural origin, can be beneficial in medical applications as they are approved by the US Food and Drug Administration and are considered safe for drug delivery, tissue engineering, wound healing, and implants. In order to successfully target and improve drug absorption, the use of chitosan and gelatin is a great example. Especially toward the central nervous system (CNS), the controlled changes in the chitosan and its derivatives have been in the scope of research for implementation as a treatment for several neurological disorders, namely, Parkinson’s and Alzheimer’s diseases. Due to the fact that chitosan is able to infiltrate the blood-brain barrier, it is especially useful for brain-aimed treatments. Among its other properties is the ability to release the drug in a controlled manner, adhere to mucus linings, and open tight junctions [124]. Using a multicomponent approach, they can be used as a platform for different particles to broaden the range of their applications. These types of materials were developed by Filip et al. [125] where they manufactured bio-nanocomposite films on a chitosan/gelatin matrix, containing Fe_3_O_4_ and ZnO nanoparticles. By the solvent casting method, they manufactured a series of different composites by in situ incorporation of inorganic nanoparticle fillers ZnO and Fe_3_O_4_ in different ratios. They concluded that the studied bio-nanocomposite samples are non-hemolytic, and the increased amounts of NPs determined slightly high values of hemolysis but within the allowed limits, confirming the low risk of thrombosis, which recommends these materials for applications in the biomedical field.

A similar approach was also implemented by Jarquin-Yáñez et al. [126] with the manufacturing of a cellulose-chitosan-nanohydroxyapatite composite for biomedical applications. However, such a complex system still needs further work, especially to carry out studies related to the effect of such composites on the differentiation of mesenchymal cells to osteoblasts in terms of bio-efficiency. The combination of two different nanosized fillers is described by Shuai et al. [127] where carbon nanotubes (CNT) and montmorillonite (MMT) a well-known material of different geometry were implemented into a biopolymer matrix. Grafted CNTs are to be inserted into the MMT interlayers in an attempt to lower the CNT aggregation, thusly improving the dispersion level. It is obtained through the ion exchange reaction, combining the two-dimensional lamellar structure of MMT with the single-dimensional tubular shape of the CNT. A series of composites was manufactured by the Authors in order to develop a PLLA scaffold. It was noticed that scaffold material containing 9% MMT-CNT had improved mechanical properties. What is also worth mentioning is the change in degradation time, which was noticeably faster, along with improved hydrophilicity, in comparison with the neat PLLA scaffold structure. Furthermore, better cell affinity was also suggested as the investigated cells were to adhere to the developed material in a more favorable fashion.

**Figure 8:**
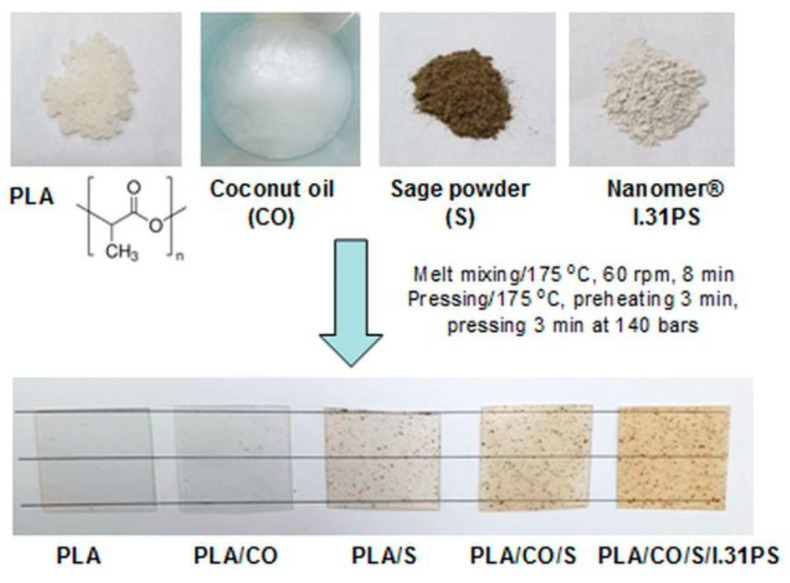
Schematic representation of the developed bioactive hybrid formulations and the visual aspects of the produced films. Reprinted with permission from [Darie-Nita R.N.] (Bioactive and Physico-Chemical Assessment of Innovative Poly(Lactic Acid)-Based Biocomposites Containing Sage, Coconut Oil, and Modified Nanoclay); published by MDPI (2023) [128].

Darie-Nita R.N. et al. [128] developed bioactive hybrid formulations by melt processing of PLA plasticized by edible coconut oil, loaded with a medicinal plant (sage) and an organo-modified montmorillonite nanoclay (Figure 8). Innovative hybrid materials could be used as bioactive materials in medical applications because, by optimizing the components, they possess flexibility, both antioxidant and antimicrobial activity, as well as a high degree of cytocompatibility, being able to induce cell adhesion and proliferation on their surface.

Biohybrid materials hold great promise as tissue scaffolds due to their unique ability to regulate cell behavior and promote cell proliferation [129]. One notable example involves encapsulating NIH/3T3 fibroblast cells within porous three-dimensional (3D) PEG-based hydrogel patches, designed to facilitate vascular regeneration [130]. These patches not only protect the cells but also incorporate a microchannel design to enhance the secretion of vascular endothelial growth factor (VEGF), which is beneficial for blood vessel formation. In another example, biodegradable collagen was used to create biohybrid microcapsules that carry mesenchymal stromal cells. These biohybrid microcapsules demonstrated the ability to improve cardiac function in a murine model when administered through intramyocardial injection.

Porous scaffolds are very important templates for cell growth and tissue development [131]. In view of magnetically guided tissue development, Tampieri et al. [132] synthesized magnetic biohybrid porous scaffolds, nucleating nano-apatite in situ on self-assembling collagen, in the presence of magnetite nanoparticles (MNPs). The implant biocompatibility was enhanced by this innovative approach as MNPs became an internal component of the scaffold.

Silica-based hybrids possess various multifunctionalities and are highly used in regenerative medicine, being approved by the FDA for human trials [133]. Due to the presence of Si–OH groups on its surface, silica nanoparticles can be easily functionalized with different materials, biomolecules, and targeting ligands. Function of the materials requirements, the interaction between silica and the organic component can be either weak (drug delivery) or strong (tissue engineering) [134].

Research on silica-based aerogels is important in the progress of silica-based materials for bone regeneration [135]. Maleki et al. proposed a new one-pot synthesis method for obtaining silica-silk fibroin hybrid aerogels with controlled pore sizes and superior mechanical strength, features that mimic the bone’s natural structure, providing also suitable conditions for bone cell growth and migration [136]. The cytocompatibility that ensured the material’s safety for use in contact with living cells, non-hemolytic nature, and ability of these silica-silk fibroin hybrid aerogels to promote osteoblast cell growth and new bone tissue formation was confirmed by in vitro and in vivo studies, proving their potential as bioactive and osteoconductive scaffolds for bone regeneration.

The incorporation of calcium phosphates within silica-biopolymer hydrogel networks is a great strategy for the development of implants with fascinating biological features, highly desired in emerging biomedical applications, particularly in bone tissue engineering. Silica-chitosan-tricalcium phosphate (TCP) xerogels (with varying contents of each component were synthesized by a sol-gel process assisted by an ultrasound probe, enabling precise modulation of the material’s properties [137]. Variations in the washing solvent (ethanol or water) and TCP content were evaluated on their influence over the xerogels’ biodegradation, in vitro bioactivity, and osteoconduction. The ethanol-washed samples containing calcium and phosphate allowed the release of both calcium and silicon ions in vitro, while only silicon was released by the water-washed samples. All hybrid materials exhibited in vitro bioactivity and improved cell growth in simulated body fluid.

**Figure 9:**
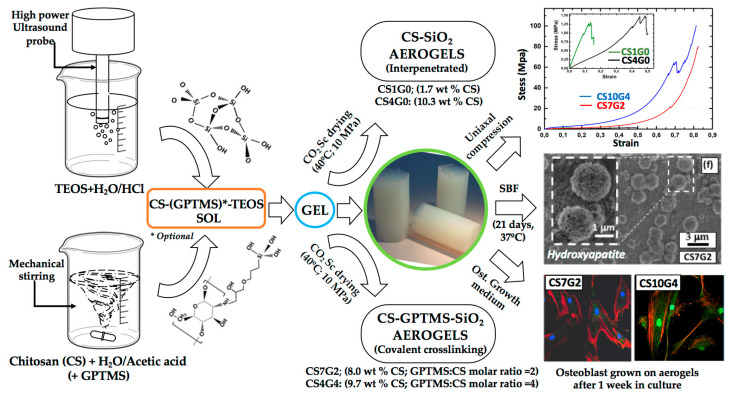
Synthesis and main features of chitosan (CS)-silica hybrid aerogels. Reprinted with permission from [Reyes-Peces et al.] (Chitosan-GPTMS-Silica Hybrid Mesoporous Aerogels for Bone Tissue Engineering); published by MDPI (2020) [138].

Aiming for tissue engineering applications, homogeneous chitosan (CS)-silica hybrid aerogels with CS contents up to 10 wt% were realized using 3-glycidoxypropyl trimethoxysilane (GPTMS) as coupling agent by the sol-gel process followed by CO_2_ supercritical drying (Figure 9). The developed materials exhibited bulk densities between 0.17 g/cm^3^ to 0.38 g/cm^3^, interconnected mesopore network with decreasing specific surface areas (1230–700 m^2^/g) and pore sizes (11.1–8.7 nm) by increasing GPTMS content (2–4 molar ratio GPTMS:CS monomer), leading to fast swelling in PBS solution. The covalent crosslinked hybrid structure was confirmed by FTIR and by the increase of four hundred-fold or more in the compressive strength up to 96 MPa. The samples synthesized without GPTMS had a weak structure consisting of interpenetrated polymer networks, with a low compressive strength of only 0.10–0.26 MPa. The new hybrid aerogels showed bioactivity in simulated body fluid (SBF), and a non-cytotoxic effect on HOB^®^ osteoblasts [138].

Utilizing sol-gel and freeze-drying techniques, Demeyer et al. incorporated mangiferin, a bioactive plant compound, into chitosan-silica hybrid nanocomposite scaffolds [139]. The study evaluated the influence of a 3D crosslinked network with the addition of ZnO nanoparticles on the physicochemical and mechanical properties of the scaffolds and concluded that the innovative approach led to tailored properties highly desirable for bone tissue engineering applications, such as porosity, fluid uptake, morphology, thermal stability, mechanical strength, biomineralization, and cell viability.

## 5. Sensors

Many sensors designed for fire and water alarm applications typically lack wet strength [140] or environmental stability, and some utilize toxic solvents during their production. Therefore, developing an easy and environmentally friendly method to create dual-function sensors that provide highly sensitive responses to both fire and water, while also ensuring excellent stability, is essential for improving safety and sustainability in our environments.

**Figure 10:**
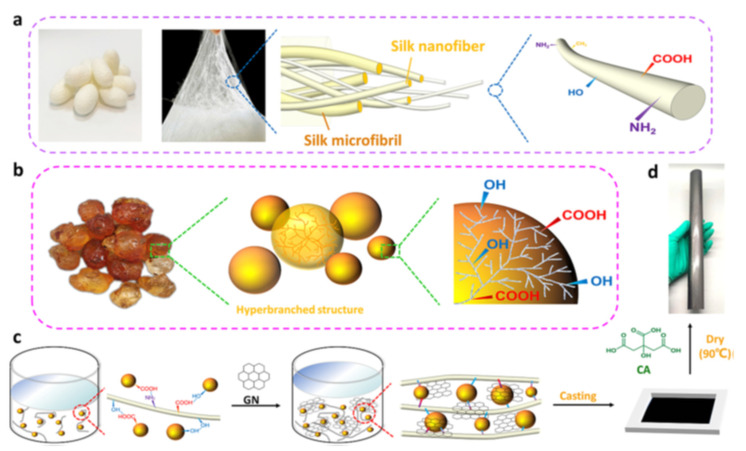
(**a**) Scheme of the hierarchical structure of the silk fibers. (**b**) PGP structure. (**c**) Preparation steps for the development of PSCG bio-films. (**d**) PSCG bio-films. Reprinted with permission from Elsevier 2024 [141].

An innovative bio-based sensor that rapidly responds to fire, water, and high temperatures while offering excellent flame retardation, wet strength, and environmental stability was developed, consisting of peach gum polysaccharide (PGP), silk nanofibers (SNF), citric acid (CA), and graphene (GN) [141]. CA, a non-toxic crosslinking agent between PGP and SNF, enhances structural stability, water resistance, and mechanical properties, while GN improves electrical conductivity and flame-retardant properties. SNF was extracted from Bombyx mori silk fibers using degumming and high-speed shearing (Figure 10a). PGP was derived from crude PG via thermal hydrolysis (Figure 10b). Flexible self-assembled PSCG biofilms were created through evaporation (Figure 10c,d). The impressive flame-retardant properties of PSCG bio-films arise from the synergy of CA and SNF, along with GN, which offers physical shielding. These films demonstrate excellent reversible and sensitive responses between 50 and 150 °C. The PSCG bio-film sensors demonstrate remarkable performance with rapid responses to flames, temperature, and water sensitivity, making them ideal for fire early warning systems and health monitoring applications.

**Figure 11:**
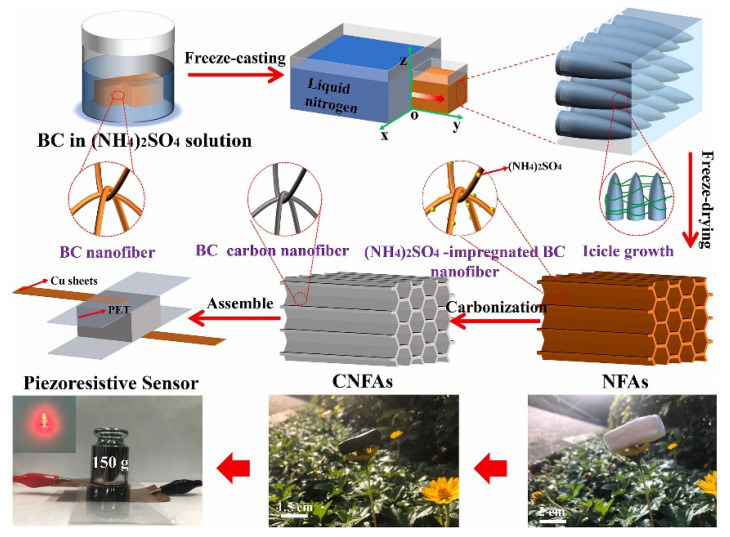
Schematic illustration of the fabrication process and photographs of NFAs, CNFAs, and a CNFA-based piezoresistive sensor. Reprinted from [147] with permission from Elsevier 2024.

Aerogels exhibit electrical conductivity, making them advantageous for developing piezoresistive sensors [142,143]. Carbon-based aerogels are especially suitable for electronic materials. Carbon aerogels are suitable for electronic materials, due to their structural controllability and remarkable electrical conductivity [144]. Their internal conductive network can create more contact points when they are deformed, resulting in higher sensitivity of the sensor [145]. Meanwhile, the 3D conductive structure can withstand large deformation, thus greatly improving the pressure range of the sensor. However, in terms of bio-based, environmentally friendly polymers, aerogel sensors are mostly obtained by using cellulose and its derivatives. This is because cellulose is widely available in the environment and relatively easy to fabricate. The basic methods used for obtaining cellulose nanofiber-based aerogels are applying high-temperature processes such as pyrolysis [146,147] in order to greatly decrease the electrical resistance, thus improving the overall electrical conductivity, and in order to minimize the negative impact of decreased material impact strength, low-temperature techniques are needed such as directional freeze drying before the carbonization [148]. The preparation scheme is illustrated in Figure 11, where the authors impregnated the structure with (NH_4_)_2_SO_4_ solution, and the following steps involved unidirectional freeze-casting, vacuum freeze drying, and carbonization.

Li et al. [149] proposed an aerogel, cellulose fiber sensor prepared in a similar manner. They proposed a strategy for the fabrication of a compressible carbonized cellulose fiber network (CCNF) with the possible application as a flexible pressure sensor obtained from wood-derived cellulose fibers. In order to avoid using TEMPO oxidation, they introduced periodate oxidation for introducing carbon groups. The into-hydrogels transformation of the cellulose fiber suspension network can be conducted by the introduction of the dialdehyde groups directly. The described method is able to guarantee success without secondary treatments needed, and it leaves the structure of the cellulose fiber network (CNF) intact after post-pyrolysis exposure to high temperature. A wearable sensor was developed that can be characterized by a detection limit of 0.47 Pa, response time of 50/20 ms for loading/unloading 50 Pa of pressure, and, also for 20 kPa, a loading/unloading pressure able to withstand more than 1000 cycles. There are also attempts to combine carbon-based aerogels with cellulose fiber. As proposed by Muthuraj et al. [150], the development of a cellulose nanofiber and carbon nanotube (CNT) aerogel via the freeze-drying technique is possible. In conclusion, the addition of CNTs into a CNF aerogel resulted in a slight increase in both the thermal conductivity and density. The suggested process is interesting as a relatively cheap technique to obtain such materials; however, it needs further investigation in order to upgrade their properties.

## 6. Water Purification

Wastewater from domestic, industrial, and agricultural activities poses a significant environmental threat. Often contaminated with hazardous organic substances like dyes, pesticides, and pharmaceuticals, as well as inorganic compounds such as heavy metals and radionuclides, this effluent can be highly toxic and resistant to degradation. Therefore, it is crucial to develop sorbents with high sorption capacity and efficiency to effectively remove these pollutants and protect our ecosystems and public health.

Dye pollutants from the textile and printing industries pose serious risks to human health and aquatic ecosystems. Organic dyes in wastewater block sunlight, hindering the metabolism and photosynthesis of aquatic plants and microorganisms. Many of these dyes are also carcinogenic and highly toxic, as seen in studies showing the harmful effects of methylene blue (MB) on human skin and the nervous system [151]. Effectively removing these harmful compounds from wastewater is crucial for safeguarding health and the environment. While various treatment methods exist, adsorption stands out for its efficiency and scalability. However, regenerating adsorbents for reuse remains a challenge. There is an urgent need for recyclable, effective, and biodegradable materials to address this issue and ensure safe water for all.

The presence of exchangeable cations in the interlayer structure gives MMT a strong cation exchange capacity, making it an excellent choice for effective cation adsorption.

**Figure 12:**
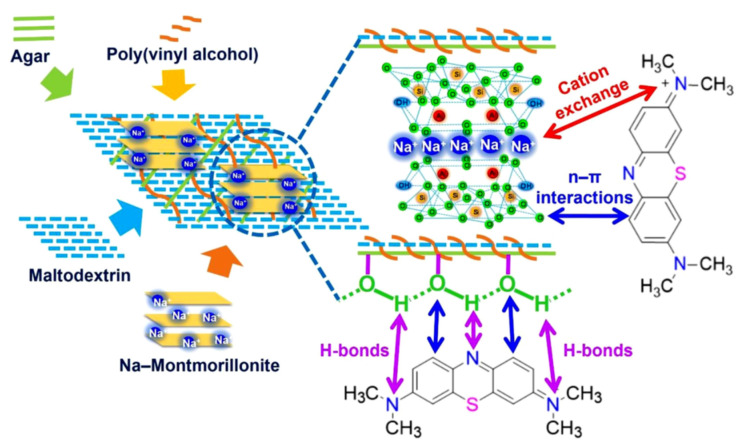
Mechanisms of MB adsorption on agar/maltodextrin/PVA MMT membrane. Reprinted with permission from Elsevier 2024 [152].

In this context, Nguyen et al. employed a casting method to prepare an agar/maltodextrin/poly(vinyl alcohol)-walled montmorillonite (AMP-MMT) composite membrane. These membranes incorporating 0–30% (*w*/*v*) MMT demonstrated a high uptake capacity of 71.51 mg/g, excellent recyclability (effective for at least five cycles), and facilitated easy desorption with sustainable eluent made from a mixture of water and ethanol [152]. The potential mechanisms for MB adsorption on an AMP-MMT hybrid membrane are displayed in Figure 12. Sodium montmorillonite clay effectively exchanges sodium cations with MB, facilitating the capture of dye molecules, as supported by other studies [153]. The lone pairs of oxygen atoms on MMT interact with the π electrons of the dye’s aromatic rings, resulting in significant n-π interactions. The hydroxyl groups from agar, maltodextrin, and PVA present on the AMP membrane create a network of intermolecular and intramolecular hydrogen bonds that interact with nitrogen atoms in MB [154]. In summary, cation exchange, hydrogen bonding, and n-π interactions all contribute to the efficient adsorption of MB onto the AMP-MMT composite.

Abou Taleb used γ-rays irradiation as an initiator to prepare hybrid nanocomposite calcium alginate/organophilic MMT, further used as an adsorbent for the removal of textile dyes acid green B and direct pink 3B [155]. Moringa oleifera seed protein-MMT material was developed by Mi et al. by employing the impregnation method [156]. The removal of water-soluble reactive red 2 (RR-2) from artificial wastewater in the batch system was evaluated, concluding that the adsorption process was influenced by several factors, including contact time, pH, the presence of inorganic salts, and initial dye concentration, demonstrating its alignment with pseudo-second-order kinetics. The authors found that the adsorption of RR-2 decreases from 7.60 mg/g to 5.92 mg/g as pH increases from 3.2 to 9.1, while it rises from 15.2 mg/g to 17.1 mg/g with a NaCl concentration increase from 0 to 30 g/L.

The freezing-thawing technique was used by Hosseinzadeh et al. [157] to prepare a novel kappa carrageenan/PVA nanocomposite hydrogel containing sodium montmorillonite nanoclay, which exhibited good adsorption capacity toward cationic crystal violet dye and the maximum adsorption of 151 mg/g. The incorporation of MMT reduced the swelling capacity of nanocomposites from 1200% to 320% due to the crosslinking effect of MMT nanoclay.

Carbon nanotubes (CNTs) are highly effective adsorbents for removing various wastewater contaminants. Their high surface area, customizable surface chemistry, and exceptional adsorption capacity make them essential for water treatment applications. Regenerating and reusing adsorbents, such as CNTs, is essential for achieving sustainable and cost-effective wastewater treatment processes. The capability of CNTs for efficient regeneration is imperative, as it directly ensures the maintenance of their adsorption capacity throughout multiple cycles. The growing demand for environmentally sustainable alternative modifications of CNTs has led to innovative approaches, such as using plant-based materials. Yadav et al. demonstrated the potential of utilizing Saccharum munja plant biomass to functionalize CNTs, resulting in an effective biosorbent for the adsorption of methylene blue [158]. The main modes of interaction between dyes and CNTs are primarily π-π interactions and electrostatic attractions, which are crucial for promoting the adsorption of dyes onto CNT surfaces.

Metal oxide semiconductors, specifically those derived from copper (Cu), manganese (Mn), cobalt (Co), chromium (Cr), vanadium (V), titanium (Ti), bismuth (Bi), and zinc (Zn), are powerful photocatalysts that have been studied and effectively utilized for the removal of organic pollutants across various environmental matrices [159]. Sukthavorn et al. [160] developed a composite nonwoven fabric using polylactic acid (PLA) and nano-silver coated titanium dioxide (Ag/TiO_2_) to effectively degrade carbaryl insecticide in water under UV irradiation. This yellow-gold composite nonwoven exhibits similar thermal and physical properties to PLA, including fiber size and a narrow size distribution. The nonwoven consistently achieves around 40% degradation of carbaryl insecticide under UV light and can be reused for up to three cycles without losing efficiency.

Naddafiun et al. [161] developed an innovative method that incorporates nanomaterials—graphene, TiO_2_, and ZnO—into cork fibers, enhancing the removal of organophosphorus pesticides during filtration due to the cork porosity and nanomaterial properties. Their findings show that these nanoparticle-infused cork fibers are highly effective, especially at 30 °C against pesticides such as diazinon, chlorpyrifos, and carbaryl. This approach provides excellent accuracy and durability, significantly reducing the need for frequent replacements.

Lignin is an eco-friendly resource and is affordable and readily accessible. Its abundance of functional groups promotes effective adsorption, which has enabled its successful application in eliminating harmful chemical pollutants from polluted water sources. Innovative adsorbents for Methyl Blue (MB) and Basic Fuchsine (BF) were developed by combining xanthan (XG) or esterified xanthan (XGAC) with Ferrite-Lignoboost^®^ lignin (CFLB) and ferrite-organosolv lignin (CFLO) hybrids [162]. The complex characterization showed that unmodified xanthan adsorbents exhibit a superior water sorption capacity when compared to their esterified xanthan counterparts. Ferrite-Lignoboost^®^ lignin and ferrite-organosolv lignin hybrid materials (CFLB and CFLO) exhibited the highest adsorption capacities for MB dye, with values of 44.73 mg/g and 37.54 mg/g, respectively. In contrast, the adsorbents based on xanthan and lignin hybrid materials (XG/CFLO and XG/CFLB) retained the largest amounts of BF dye, measuring 36.23 mg/g and 33.33 mg/g, respectively.

Jiao et al. [163] developed magnesium oxide functional group-loaded lignin-based biochar (MFLC) using a two-step carbonization process. The maximum adsorption capacity of this biochar for phosphates reached as high as 906.82 mg g^−1^. This innovative preparation method creates a significant number of well-dispersed, small magnesium oxide nanoparticles on the surface of the MFLC, which greatly enhances its adsorption activity. Furthermore, the structure of the lignin gel helps to prevent lignin aggregation, allowing for better exposure of the aromatic rings and functional groups. This increases the number of contact sites, making it more effective in adsorbing pollutants.

**Figure 13:**
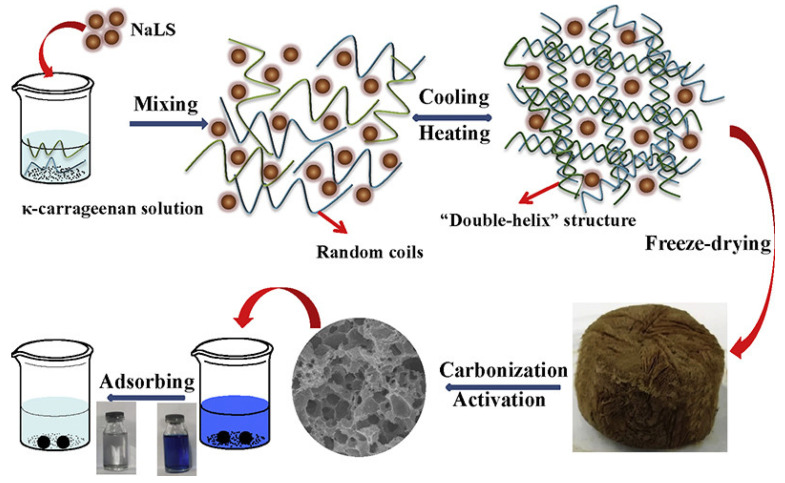
Schematic description of obtaining carbon aerogels derived from sodium lignin sulfonate embedded in carrageenan skeleton for methylene blue removal. Reprinted with permission from Elsevier 2024 [164].

Lv et al. [164] proposed an eco-friendly and cost-effective method for the facile preparation of lignin carbon aerogels using carrageenan as a three-dimensional skeleton green. The carbon aerogels obtained after 30 min of KOH activation exhibited an interconnected three-dimensional network, featuring a hierarchical porous structure with micropores, mesopores, and macropores (Figure 13). Its surface area reached 594.6 m^2^/g and the adsorption of methylene blue was up to 421.94 mg g^−1^. This innovative study addresses the recycling issues related to sulfite pulping wastewater and dye wastewater.

## 7. Electromagnetic Interference (EMI) Shielding

Nowadays, electromagnetic energy radiation pollution has become the fourth largest pollution after noise pollution, air pollution, and water pollution, which can even seriously disturb the ecosystem and affect human health [165].

EMI shielding has proven to be most important in several industries to counteract the harmful impact of EMI on electronic devices, delicate installations, and, not least, people. Due to their non-biodegradability and energy-consuming manufacturing steps, traditional EMI shielding materials, mostly composed of metals and metal alloys, raise ecological issues. In this context, the demand for environmentally friendly materials for EMI shielding applications is increasing.

Among the potential green candidates for reducing electromagnetic (EM) issues in next-generation electronic devices, one could mention biopolymers such as cellulose, PLA, and starch due to their features, for instance, high aspect ratio, flexibility, lightweight, high mechanical strength, thermal stability, and tunable microwave absorption to the electromagnetic interference (EMI) shielding composites [166]. Some other types of EMI shielding sustainable materials are derived from bamboo, wood, or industrial recycled materials [167].

Innovative eco-friendly EMI shielding materials were created by the use of recycled polymer waste. Hybrid materials were developed by using crosslinked polyethylene (CLPE) cable waste and CNT filler; the flexible composites exhibited an EMI SE of 35 dB with great mechanical properties such as a tensile strength over 20 MPa and elongation at break of 280% [168].

**Figure 14:**
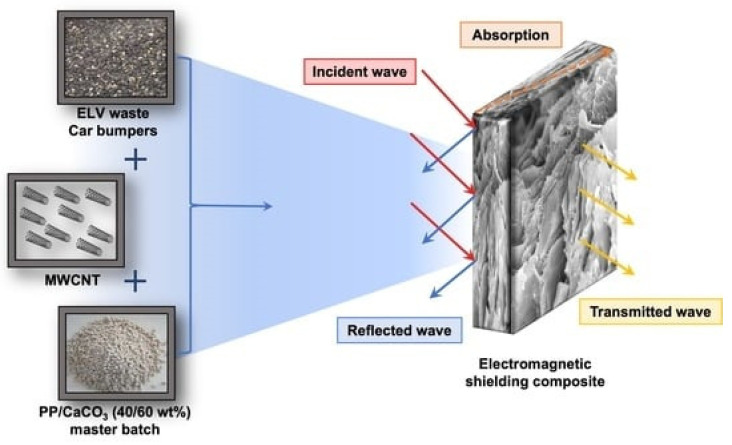
Development of an efficient sustainable EMI shield containing recycled EPDM from waste car bumper, a masterbatch of 40PP/60CaCO_3_, and a conductive CNT nanofiller. Reprinted with permission from [Moaref et al.] (From Waste to Value Added Products: Manufacturing High Electromagnetic Interference Shielding Composite from End-of-Life Vehicle (ELV) Waste); published by MDPI (2024) [170].

Reutilization of recycled plastic from end-of-life vehicles (ELVs) could contribute to an eco-friendly environment and sustainable development [169]. In some cases, the recycled plastic from ELVs could be reintroduced in the production of EMI shields with high absorption that might be used for protecting electronic devices in vehicles. Moaref et al. melt-mixed recycled waste bumpers of ethylene propylene diene monomer (EPDM) rubber from ELVs with a masterbatch of 40PP/60CaCO_3_ and a conductive CNT nanofiller. The resulting low-cost and lightweight hybrid nanocomposite showed an electrical conductivity of 5.2 × 10^−1^ S⋅cm^−1^ for 5 vol% CNT in a 30 wt% EPDM/70 wt% PP/CaCO_3_ masterbatch, with a great EMI shielding effectiveness of 30.4 dB (Figure 14). The authors demonstrated the efficacy of waste material reuse, which increased the strain at the break by 54% and the yield strain by approximately 46% relative to the same composite without waste [170].

It is important to mention, though, that the potential impacts of carbon nanotubes (CNTs) on the environment and human health demand immediate attention. Adverse effects stem from variations in their structure, size, shape, surface charge, and contaminants, increasing the risk of their entry into the human body through inhalation or the food chain. CNTs can negatively interact with critical biomolecules like DNA, RNA, proteins, and enzymes, posing significant threats to aquatic life [171]. Therefore, it is crucial to explore safe disposal methods and develop biocompatible CNTs while conducting thorough research on the hazardous effects of modified CNTs on ecosystems and human health.

An effective strategy for the sustainable development of high-performance green EMI shielding materials was proposed by Uddin et al. [172] who used a facile, green, and low-cost procedure through the simple soaking and carbonization process to realize a waste tissue-paper-derived cellulose carbon (WTCC)/molybdenum disulfide (MoS_2_) composite using WTCC from waste napkin papers collected from local office bins. MoS_2_ nanosheets have a large surface area and act as a bridge between the cellulose fibers, being anchored by functional groups on the surface of the cellulose, resulting in an electrically conductive network highly required for improved shielding. The innovative hybrid composites showed environmentally friendly shielding features such as microwave absorption of about 15 dB and total shielding effectiveness of 28 dB by modulating polarization-dependent losses, effectively suppressing secondary electromagnetic pollution.

Biomass-based sustainable shielding materials could contain a main biomass component sourced from husks, leaves, peels, shells, or other non-wood/non-cellulose products that might be in biochar, carbon aerogel, and composites of more than one type of sustainable material [167,173]. Another approach for the development of sustainable shielding materials was developed by Gu et al. who initially carbonized at 800 °C sustainably sourced shaddock peel using low-toxicity reagents in freeze-drying peels and further incorporated 20 wt% Shaddock peel-derived carbon aerogel into paraffin wax, obtaining impressive properties such as a SE R of 29.50 dB and an effective absorption bandwidth spanning 5.80 GHz with a relatively thin thickness of only 1.7 mm. The integrated 3D porous carbon network leads to many internal reflections, conductive loss, polarization loss, and dielectric loss [174].

A study established that combining carbon nanotubes (CNTs) with rice husk creates a composite material that absorbs microwave signals in the 12.4 to 18 GHz range. The optimal absorption performance is achieved with 5% CNTs, making this composite promising for microwave absorption and shielding applications [175].

In view of using greener materials and processes, biodegradable polymers can incorporate bio-based carbons resulting in accurately sustainable and environmentally friendly EMI shielding hybrid composites. Facile melt extrusion at 190 °C and subsequent hot-pressing methods were used to design and process environmentally friendly and biodegradable two- and three-phase composites comprising 30 wt% graphite, 20 wt% biochar derived from pine chips and PLA that could be used as very efficient EMI shielding materials for applications in the K-band frequency range of 18–26.5 GHz, important for 5G telecommunication [176]. The new lightweight and easy-to-design composites showed high conductivity over 30 S/m with staggering shielding effectiveness over 30 dB for very thin films of 0.25 mm thickness. Microwave measurements have shown adequate scattering of electromagnetic waves on fillers and, consequently, their enhanced travel distances in the absorbing medium play an important role in attenuation. The demonstrated properties showed that the developed environmentally friendly and biodegradable hybrid composites are promising for applications of wearable and portable devices, and radiation-sensitive electronics and sensors, with potential in aeronautics, astronautics, and robotics, where dimensions and weight are very important as well.

Aerogels could also be considered to construct hybrid materials with low density and efficient dissipation of electromagnetic energy, although achieving performant aerogels with good EMI shielding or EMW absorption performance and acceptable mechanical properties is quite difficult. Han et al. reported the development of various aerogels based on biopolymers such as cellulose, lignin, chitin/chitosan, and alginate, offering high-efficiency EMW shielding and absorbing properties [177].

## 8. Conclusions and Future Challenges

The development of environmentally friendly hybrid polymeric materials to substitute petroleum-derived plastics used in various applications is an important approach to being responsible for the sustainable development of society.

Hybrid polymeric materials that exploit the synergy between natural fillers and biopolymeric matrices can lead to enhanced properties along with maintaining environmental considerations. However, creating new hybrid polymeric formulations and technologies often requires specialized raw materials that may not be widely available or commercially viable, potentially complicating the supply chain dynamics. In order to overcome these challenges, it is highly desired to establish strategic partnerships, diversify sourcing options, and invest in alternative raw materials, preferably environmentally friendly. Fulfilling these stages will guarantee continuity and sustainability in the supply chain.

Nanoparticles such as titanium dioxide, silver, nanoplastics, copper oxide, etc., or nanotubes like CNT and others are highly useful due to their functional properties, but they are also known to pose a significant concern in various applications due to their potential health risks and environmental impact as they can induce oxidative stress, inflammation, genotoxicity, and carcinogenic effects. Mitigation solutions such as their modification and inclusion in valuable materials, advanced analytical techniques, and sensitive sensors for NP detection for safeguarding public health and policy implementations are continuously developing.

Collaborative multidisciplinary approaches are vital for thoroughly evaluating the efficacy, bioavailability, and pharmacokinetics of newly developed hybrid polymeric multipurpose systems. This is particularly important due to the limited availability of animal models needed to test diverse novel materials for targeted medical applications. Engaging experts from various fields will ensure a comprehensive understanding and more reliable results in pursuing these innovative solutions.

Sustainable hybrid materials containing bamboo, wood, cellulose, PLA, and recycled materials face significant challenges in performance and durability compared to traditional metal-based options in EMI applications. Structural changes due to variations in time and temperature can lead to defects, which affect electromagnetic interference (EMI) shielding reliability. Nevertheless, there are extensive opportunities for innovation in sustainable EMI shielding. Researchers and industry professionals should actively explore new materials and advanced fabrication techniques to enhance performance and durability. This interdisciplinary approach encourages collaboration among materials science, engineering, and environmental science. Using renewable resources and reducing reliance on non-biodegradable materials can effectively support environmental sustainability and minimize the carbon footprint of shielding technologies.

Life cycle assessments are highly necessary in innovative studies involving newly developed sustainable hybrid materials; therefore, primary data collection to build eco-profiles of innovative materials should be carefully included in current databases.

The selection and fabrication of new sustainable hybrid polymeric materials sourced from raw and renewable origins can combine the development of hybrid materials with green manufacturing, and this could be improved through advanced artificial intelligence methods. Such sustainable production of materials through intelligent hybridization and eco-efficient, digital manufacturing can enable a long-term circular economy.

Research and development concerning hybrid polymeric materials should be further developed through cooperation between industry, academic, and government agencies, fueling the act of implementation into practical applications. Interdisciplinary and collaborative endeavors are crucial for solving problems that can emerge in the future with the tackling of difficult challenges from climate change to environmental pollution that stand before society today. Therefore, future breakthroughs are expected in the field of polymer technology and materials science via a more sustainable and environmentally friendly approach.

Given the increasing progress in materials science, cell biology, electronics, and so on, it is expected that environmentally friendly hybrid polymeric materials will continue to expand the frontiers of the biomedical sciences and engineering.

## Data Availability

Data are contained within the article.
